# Inpatient addiction care is associated with increased vaccinations, medication for opioid use disorder and naloxone prescribing among patients with infective endocarditis in a rural state

**DOI:** 10.1186/s13722-025-00614-6

**Published:** 2025-10-16

**Authors:** Eva J. Farkas, Victoria Molina, Brittany Mohoney, Wendy Craig, Jessie Schaumberg, Amy McAuliffe, Kinna Thakarar

**Affiliations:** 1 Tufts University School of Medicine, 145 Harrison Avenue, Boston, MA 02111 USA; 2 MaineHealth Maine Medical Center Portland, 22 Bramhall St., Portland, ME 04102 USA; 3 Advocate Aurora Health, 2845 Greenbrier Road, Green Bay, WI 54311 USA; 4 MaineHealth Institute for Research, 81 Research Drive, Scarborough, ME 04074 USA

**Keywords:** Rural, Multidisciplinary, Inpatient, Infectious disease

## Abstract

**Background:**

Rural states have experienced increasing injection drug use (IDU)-associated infective endocarditis (IE). Inpatient addiction consult services can reduce morbidity associated with substance use and other infectious complications, such as IDU-IE. However data on the impact of such services on healthcare utilization are limited, particularly in rural communities.

**Methods:**

This retrospective study assesses clinical and health service utilization data from index hospitalizations for IDU-IE before and after the implementation of the Integrated Medication for Addiction Treatment (IMAT) program at a tertiary care center in a rural state. We summarized data descriptively, stratified by both pre- and post-IMAT program implementation and IDU-IE and non-IDU IE. We also performed exploratory multivariable analyses assessing the association between IMAT program implementation and various outcomes. The primary outcomes were: 1) 90-day emergency department (ED) visits and 2) 30-day hospital readmissions post-discharge. Secondary outcomes included prescriptions at time of discharge for medication for opioid use disorder (MOUD), naloxone and key vaccinations.

**Results:**

We identified *n* = 99 patients with IDU-IE. Comparing pre- and post-IMAT implementation, 30-day readmissions trended lower post-IMAT (18%) versus pre-IMAT (22%), although the difference was not significant (*p* = 0.7). 90-day ED visits remained stable (37%, *p* > 0.9). The proportion of MOUD prescribing (24% versus 80%), hepatitis B vaccination (29% versus 51%), and Tdap vaccination (7.3% versus 41%) increased significantly following IMAT implementation (*p* < 0.001, *p* = 0.037 and *p* < 0.001, respectively). In a regression analysis controlling for age, housing status, primary care provider, age, hepatitis C, cardiac device, Duke’s criteria, valve affected, alcohol use disorder, payer, and vascular or infectious complications, the IMAT program was not significantly associated with the primary outcomes or with hepatitis B vaccination. However, the IMAT program was associated with increased MOUD prescribing (aOR: 110; CI:16–1500), naloxone prescribing (aOR 18; CI: 1.1–1600) hepatitis A vaccination (aOR: 5.3; CI: 1.2–32), and Tdap vaccination (aOR: 9.2; CI: 2.0–59).

**Conclusions:**

Inpatient addiction services were associated with increased prescribing of MOUD, naloxone and key vaccinations, though the incidence of acute healthcare utilization did not change. These results highlight hospitalization as an opportunity to connect patients with IDU-IE to MOUD and preventative care, particularly in rural areas where access to such services may be limited.

**Trial registration:**

Not applicable.

**Supplementary Information:**

The online version contains supplementary material available at 10.1186/s13722-025-00614-6.

## Background

Morbidity and mortality in the drug overdose crisis arise not only from toxidrome and overdose, but also from infectious complications. These include communicable infectious diseases (including human immunodeficiency virus [HIV], Hepatitis A [HAV], Hepatitis B [HBV] and Hepatitis C [HCV]), but also serious infections requiring extensive courses of parenteral antibiotics [1]. These latter infectious sequelae of drug use include tetanus, cotton fever, cellulitis, osteomyelitis, bacteremia, and infective endocarditis (IE) [2]. Even as nationwide fatalities due to overdose are slowly declining nationwide, thought in part to be due to the increasing prevalence and accessibility of naloxone in the community, the incidence of IE associated with injection drug use (IDU-IE) is increasing across the United States [3–7]. One study using the National (Nationwide) Inpatient Sample (NIS) found the incidence of IDU-IE to rise from 48 per 10,000 to 79 per 10,000 in 2016 [3]. This rise in incidence is thought to be multifactorial, with underutilization of harm reduction services and lack of access to quality addiction care services thought to play a prominent role [8].

Notably, while IE not associated with IDU (non-IDU-IE) and IDU-IE both are infections of the endocardium and cardiac valves, the natural history of the two processes differ markedly. IDU-IE is more likely to affect right-sided heart valves and consequently seed septic pulmonary emboli, than non-IDU-IE [9, 10]. Additionally, the demographics, clinical characteristics and clinical outcomes of patients with IDU-IE differ from those of non-IDU-IE. Multiple studies have shown that patients with IDU-IE are younger and have fewer comorbidities than those with non-IDU-IE [3, 4, 11–13]. At the same time, patients with IDU-IE have similar or higher rates of cardiac surgery, in-hospital mortality, post-discharge emergency department (ED) visits and recurrence of IE [12, 14, 15]. Patients with IDU-IE also have longer length of stay in the hospital [16]. Together, these findings reflect increased morbidity and healthcare utilization among people with IDU-IE.

These population differences and disparate clinical outcomes, as well as the increased cost of hospitalizations for IDU-IE, have increasingly motivated health systems to identify new strategies to meet the unique medical and psychosocial needs of people with IDU-IE, including providing addiction care during patients’ hospitalizations for IE and other serious infections [17–24]. These interventions, which often include consultation by addiction medicine specialists or social workers and medication for opioid use disorder (MOUD) prescribing, are associated with increased MOUD prescribing; however data on their impact on healthcare utilization among people with IDU-IE are limited and show mixed results [25]. One study of 147 people who inject drugs (PWID) with serious infections in New Hampshire showed a decrease in post-discharge hospital readmissions and self-directed discharges among patients receiving any form of MOUD, when compared to those not receiving MOUD [23]. Another study of 70 patients with IDU-IE in Wisconsin found that the implementation of a multidisciplinary intervention to address substance use during IE hospitalizations increased MOUD prescribing, but did not change readmission rates [21]. The increasing morbidity and healthcare utilization associated with IDU-IE necessitates better understanding of the impact of addiction medicine interventions in the inpatient setting. Indeed, such understanding is necessary to reduce health care disparities between patients with IDU-IE and non-IDU-IE. Importantly, given the disproportionate impact of the drug overdose crisis on rural areas, it is critical that addiction care interventions in the acute care setting for IDU-IE are studied in rural areas [26].

In this study, we examine the association of a multidisciplinary inpatient addiction care with healthcare utilization and other preventative care outcomes among people with IDU-IE at a tertiary care center in a rural state. Additionally, we compare the demographic and clinical characteristics of patients admitted to our institution with IDU-IE before and after the implementation of the addiction care team.

## Methods

### Study design and patient population

This study is a single-center, retrospective cohort study performed at a 700-bed tertiary care facility in Portland, Maine with a large rural catchment area serving more than one million people across Maine and southern New Hampshire. The study period extended from January 1^st^, 2013 to January 1^st^ 2019, encompassing the three years preceding (01/01/2013–12/31/2015) and the three years following (01/01/2016–01/01/2019) the implementation of the IMAT program. All hospitalized patients receiving a transesophageal echocardiogram (TEE) during the study period were potentially eligible for inclusion in the study. Exclusion criteria include: patients who 1) were not admitted to the hospital at time of TEE; 2) were less than 18 years of age at the time of TEE; and 3) had no definite or possible IE by chart review. For patients hospitalized more than once with definite or possible IE over the course of the study period, only their first (index) hospitalization was included in the analysis. The independent variable of interest in our study is whether the index hospitalization occurred before or after the implementation of the IMAT program. This study was approved after expedited review by the MaineHealth Institutional Review Board.

### IMAT program

The IMAT program is a multidisciplinary addiction care team in the inpatient setting at our institution, designed to provide addiction care to patients hospitalized for management of other medical conditions. At the time of the study, the IMAT team consisted of consult-liaison psychiatrists (with and without addiction psychiatry fellowship training), advanced practice providers (psychiatric nurse practioners, physician assistants), a licensed clinical social worker and an engagement specialist. Compared to prior care provided by the psychiatry consult liasion service alone, the IMAT program introduced new interdisciplinary roles. The licensed clinical social worker provided therapy and motivational interviewing. The engagement specialist connected patients to case management, housing support and inpatient and outpatient MOUD treatment programs, per patient preference. All patients with known or suspected substance use disorders (including opioid use disorder, stimulant use disorders, alcohol use disorders and polysubstance use) and who were amenable to receiving addiction care services were eligible for evaluation and treatment by the IMAT team. When consulted by primary teams, the IMAT program involved inpatient assessments as well as inductions or maintenance of MOUD as indicated and desired by the patient. Follow-up visits with IMAT clinicians were offered at the request of patients and primary care team for further management of patients’ substance use and mental health disorders. IMAT clinicians facilitated connections to outpatient addiction care resources. The IMAT program also included optional group peer-support sessions for hospitalized patients facilitated by an IMAT clinician, although acuity of patient illness and staff availability to transport patients challenged consistent implementation. New order sets in the EHR were also developed, prompting primary teams to order appropriate withdrawal symptom scoring (i.e. Clinical Opiate Withdrawal Scale, Clinical Institute Withdrawal Assessment for Alcohol) and as-needed medications for withdrawal management (i.e. clonidine, lorazepam, or phenobarbital). These order sets also included orders for pregnancy testing, urine toxicology screening, baseline electrocardiograms, non-opioid medications for pain, key vaccinations (Hepatitis A, Hepatitis B and Tdap) and low dose, standard and high dose pathways for buprenorphine induction. Throughout the duration of the study period, multidisciplinary educational sessions on caring for people with substance use disorders were held by IMAT team members for hospital staff. These sessions highlighted the infectious sequelae of drug use and impact of vaccine-preventable illness among people with IDU.

In light of the multidisciplinary nature of hospital-based addiction care, efforts have been made to categorize various inpatient addiction medicine services. One such model, by Englander, et al. [27] delineates between interprofessional addiction consult services providing comprehensive, multidisciplinary addiction care regardless of patients’ readiness for change in substance use behaviors, and more limited hospital-based opioid treatment programs, providing MOUD to patient’s with opioid use disorder desiring medical treatment. The IMAT program presented here aligns most closely with the interprofessional addiction consult service category, although it should be stressed that participation in the IMAT program and receipt of higher level addiction care services through the IMAT was voluntary, per patients’ goals and preferences.

### Outcomes

Our primary outcomes were 90-day post-discharge ED visits and 30-day post-discharge hospital readmissions. secondary outcomes were prescriptions for MOUD and naloxone and the receipt of key vaccinations by the time of hospital discharge. Secondary outcomes included the percentage of patients without documentation of vaccination for Hepatitis A, Hepatitis B, and Tdap who received those vaccines by time of discharge, and the percentage of patients receiving MOUD and prescriptions for naloxone by the time of discharge.

### Data collection

We used our study site’s Cardiovascular Information Systems (CVIS) database to identify patients undergoing TEE and review their TEE results. Among these patients, study staff performed retrospective chart review using the electronic health record (EHR) to identify those patients with definite or possible IE using modified Duke’s criteria [28]. Study staff performed manual chart review to collect patient demographics, clinical characteristics (including substance use histories) and post-discharge health service utilization data from patients’ index hospitalizations for IE within the study period. Variable definitions for demographics and clinical characteristics have been described previously [14]. EJF additionally reviewed all collected data and discussed any discrepancies with KT and WC. 30-day hospitalizations included admission to any hospital within the health system, not just the tertiary care center. Likewise, 90-day ED visits included encounters at all EDs in the health system. Vaccination data for hepatitis A, hepatitis B and Tdap were obtained using immunization records available in the patient’s chart and, when applicable, serological studies performed during the patient’s hospitalization for IE, so that the percentage of patients requiring these vaccines (i.e. due to absence of seropositivity, or prior vaccination records) at time of admission who received them prior to discharge could be evaluated as a study outcome.

### Statistical analysis

We summarized clinical and demographic characteristics using descriptive statistics, both overall and after stratification by IDU subgroup. We compared data between subgroups using chi-square, Fisher’s exact test or Mann-Whitney U tests, as appropriate, to compare clinical and demographic characteristics between patients with IDU-IE and non-IDU-IE. Univariate analyses were used to compare clinical and demographic variables among study participants with or without a specified primary outcome. Collinearity was assessed by variance inflation factor (VIF), with non-collinear variables having VIF < 2. We assessed the unadjusted relationship between pre- or post-IMAT implementation status and outcome measures using logistic regression. All non-collinear variables that demonstrated a univariate relationship with an outcome (*p* < 0.10) were then included as covariates in a logistic regression model to assess the adjusted relationship between pre- or post-IMAT status and the primary outcomes. Age was also included due to its clinical relevance. The covariates that were ultimately included were age, housing status, whether or not the patient had a documented primary care provider, known history of hepatitis C infection, presence of a cardiac device, whether IE was classified as “definite” or “possible” by Duke’s criteria, whether a mechanical or native heart valve was involved, the presence of comorbid alcohol use disorder, having insurance through a public payer, and the presence of any extra-cardiac vascular or infectious complications of IE. Significance was accepted at *p* < 0.05. Given that survival to 90 days following discharge from the index IE hospitalization was required to assess the primary outcomes, patients who died of any cause during their IE admission or within 90 days of discharge were excluded from the regression analyses. All statistical analyses were completed using RStudio (version 2023.06.1).

## Results

### Differences in demographic and clinical characteristics in patients with IDU-IE and non-IDU-IE

Among the 289 hospitalized patients undergoing TEE during the study period; *n* = 193 (67%) were found to have possible or definite IE by Duke’s criteria and were stratified based on having IDU-IE (*n* = 99) or non-IDU-IE (*n* = 94). Compared with the non-IDU IE group, participants with IDU-IE were significantly more likely to be publicly insured (*p* < 0.001), be unhoused (*p* < 0.001), have a history of any mental health condition (*p* < 0.001), have a history of hepatitis C infection (*p* < 0.001), and were less likely to have a primary care provider (PCP) (*p* < 0.001) than patients with non-IDU-IE (SI Table A). No patients with IDU-IE who survived to discharge experienced all-cause mortality in the 90 days immediately following discharge, while 5 patients with non-IDU-IE died within 90 days (SI Table B). However, patients with IDU-IE were more likely to experience vascular complications of IE (*p* < 0.001) and infectious complications of IE (*p* < 0.001) (SI Table B).

### Population differences before and after IMAT program implementation

We also compared the demographic (Table 1) and clinical (Table 2) characteristics of patients with IDU-IE in the three years preceding and the three years following the implementation of the IMAT program. The pre-IMAT (*n* = 42) and post-IMAT (*n* = 57) patients with IDU-IE differed only with respect to the prevalence of alcohol use (33% pre-IMAT, 16% post-IMAT, *p* = 0.041) and prevalence of native valve over prosthetic valve endocarditis (79% pre-IMAT, 93% post-IMAT, *p* = 0.036).

**Table 1:** Demographic and health characteristics of patients with injection drug use-associated infective endocarditis at Maine Medical Center before and after IMAT implementation

Characteristic	**Overall** ^*1*^	**Pre-IMAT** ^*1*^	**Post-IMAT** ^*1*^	**p-value** ^*2*^
**n**	**99 (100)**	**42 (42)**	**57 (58)**	
**Assigned female at birth**	39 (39)	15 (36)	24 (42)	0.5
**Average age**	34	34	33	>0.9
**Insurance**				0.6
Public insurance only	65 (66)	27 (64)	38 (67)	
Uninsured	28 (28)	14 (33)	14 (25)	
Private insurance only	4 (4.0)	1 (2.4)	3 (5.3)	
Both public and private insurance	2 (2.0)	0 (0)	2 (3.5)	
**Documented PCP** ^*3*^	67 (68)	30 (71)	37 (65)	0.5
**Unhoused**	28 (28)	9 (21)	19 (33)	0.2
**No mental health conditions**	1 (1.0)	0 (0)	1 (1.8)	>0.9
**Documented UTD**^*4*^ **Tdap**	16 (16)	0 (0)	16 (28)	<0.001
**Documented UTD**^*4*^ **Hep A**	8 (8.1)	2 (4.8)	6 (11)	0.2
**Documented UTD**^*4*^ **Hep B**	29 (29)	9 (21)	20 (35)	0.2
**History of Hepatitis C**				0.13
Positive Hep C test^*5*^	69 (70)	31 (74)	38 (67)	
Negative Hep C test	20 (20)	5 (12)	15 (26)	
No Hep C screening done	10 (10)	6 (14)	4 (7.0)	
**Opioid use disorder**	90 (91)	37 (88)	53 (93)	0.5
**Cocaine use disorder**	30 (30)	16 (38)	14 (25)	0.15
**Cannabis use disorder**	18 (18)	11 (26)	7 (12)	0.076
**Amphetamine use disorder**	9 (9.1)	4 (9.5)	5 (8.8)	>0.9
**Comorbid alcohol use** ^*6*^	23 (23)	14 (33)	9 (16)	0.041

^*1*^ n (%)

^*2*^ Pearson’s Chi-squared, Fisher’s exact test or Mann-Whitney U test

^*3*^ Primary care provider (PCP) on file

^*4*^ Up to date

^*5*^ Viral load or serology

^*6*^ Alcohol use disorder or “alcohol misuse” documented in patient’s chart

**Table 2:** Clinical characteristics of patients with injection drug use-associated infective endocarditis at Maine Medical Center before and after IMAT implementation

Characteristic	**Overall** ^*1*^	**Post-IMAT** ^*1*^	**Pre-IMAT** ^*1*^	**p-value** ^*2*^
**n**	**99 (100)**	**57 (58)**	**42 (42)**	
**Definite Endocarditis** ^*3*^	88 (89)	52 (91)	36 (86)	0.5
**Valve type**				0.2
Right-sided	50 (51)	30 (53)	20 (48)	
Left-sided/left and right-sided	39 (39)	24 (42)	15 (36)	
No definite valvular involvement	10 (10)	3 (5.3)	7 (17)	
**Native valve endocarditis**	86 (87)	53 (93)	33 (79)	0.036
**Vascular phenomena** ^*4*^	50 (51)	26 (46)	24 (57)	0.3
**Immunological phenomena** ^*5*^	9 (9.1)	6 (11)	3 (7.1)	0.7
**Infectious complications** ^*6*^	73 (74)	41 (72)	32 (76)	0.6
**Cardiac device (i.e. pacer, automatic implantable cardioverter defibrillator)**	4 (4.0)	3 (5.3)	1 (2.4)	0.6
**Blood culture**				0.14
Gram-positive only	82 (83)	49 (86)	33 (79)	
Negative blood cultures	9 (9.1)	2 (3.5)	7 (17)	
Gram-negative only	3 (3.0)	2 (3.5)	1 (2.4)	
Polymicrobial	3 (3.0)	2 (3.5)	1 (2.4)	
Yeast only	2 (2.0)	2 (3.5)	0 (0)	

^*1*^ n (%)

^*2*^ Fisher’s exact test or Pearson’s Chi-squared test

^*3*^ Versus possible endocarditis, by Duke’s criteria

^*4*^ I.e. major arterial emboli, septic pulmonary infarcts, mycotic aneurysm, intracranial hemorrhage, conjunctival hemorrhages and/or Janeway lesions

^*5*^ I.e. glomerulonephritis, Osler nodes, Roth spots and/or rheumatoid factor

^*6*^ I.e. septic emboli, septic joint, osteomyelitis, skin/soft tissue infection and/or epidural abscess

^*7*^ I.e. pacer, automatic implantable cardioverter defibrillator

### Healthcare utilization and care quality outcomes

The univariate relationships between pre- and post-IMAT time period and post-discharge healthcare utilization and care quality outcomes are shown in Table 3. Among patients with IDU-IE, the implementation of the IMAT program did not change rates of post-discharge healthcare utilization, with respect to both the percentage of patients experiencing 30-day hospital readmissions (22% incidence pre-IMAT, 18% post-IMAT, *p* = 0.7) and 90-day ED visits (37% pre-IMAT, 37% post-IMAT, *p* > 0.9).

**Table 3:** Unadjusted healthcare utilization and care quality outcomes among patients with injection drug use-associated infective endocarditis at Maine Medical Center before and after IMAT implementation

Outcome	Pre-IMAT (n = 42)	Post-IMAT (n = 57)	**p-value** ^*1*^
**Death during admission**	12%	14%	0.5
**Readmitted within 30 days**	22%	18%	0.7
**90-day ED**^*2*^ **visits**	37%	37%	>0.9
**MOUD**^*3*^ **at discharge**	24%	80%	<0.001
Methadone	12%	22%	0.2
Buprenorphine	0%	12%	0.030
Buprenorphine/naloxone	12%	45%	<0.001
**Discharged with naloxone**	2%	14%	0.067
**Hepatitis A vaccine given**	9.8%	24%	0.069
**Hepatitis B vaccine given**	29%	51%	0.037
**Tdap given**	7%	41%	**<**0.001

^*1*^ Pearson’s Chi-squared test or Fisher’s exact test

^*2*^ Emergency Department

^*3*^ Medication for opioid use disorder

### MOUD and naloxone prescribing

Prior to the implementation of the IMAT program, 24% of patients with IDU-IE had received prescriptions for MOUD in the course of their IE hospitalization. In the three years following IMAT implementation, the rate of patients with IDU-IE receiving prescriptions for MOUD by the time of discharge rose to 80% (*p* < 0.001). A notable increase in the rate of patients with IDU-IE receiving naloxone prescriptions at discharge also increased, although the percentage of patients prescribed naloxone prescribing remained low (2% pre-IMAT, 14% post-IMAT, *p* = 0.067) (Table 3).

### Key vaccinations

The unadjusted rates of receipt of vaccinations for hepatitis A, hepatitis B, and Tetanus, Diphtheria and Pertussis (Tdap) among patients with IDU-IE and indication for vaccination increased significantly following the implementation of the IMAT program, with rates increasing for hepatitis A from 9.8% to 24% (*p* = 0.069), for hepatitis B from 29% to 51% (*p* = 0.037) and Tdap from 7.3% to 41% (*p* < 0.001) (Table 3).

### Adjusted logistic regression

**Fig. 1:**
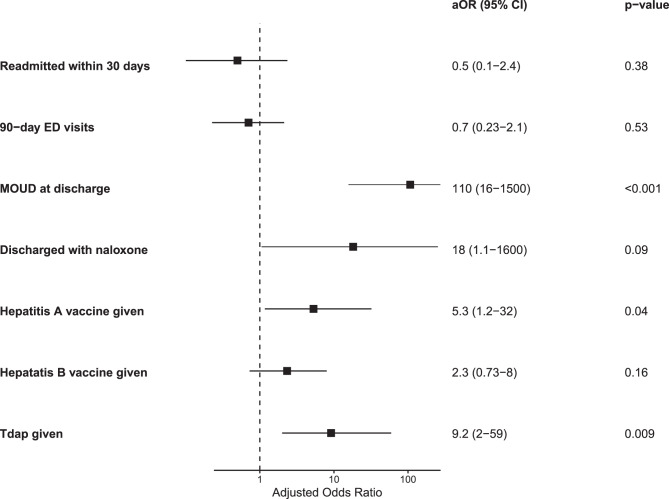
Adjusted odds ratios of primary and secondary outcomes following implementation of the IMAT program among patients with IDU-IE at MMC, 01/01/2013–01/01/2019. Covariates in the logistic regression model included age, housing status, having a documented primary care provider, history of hepatitis C infection, any cardiac device, whether IE was classified as “definite” or “possible” by Duke’s criteria, whether a mechanical or native heart valve was involved, the presence of comorbid alcohol use disorder, having insurance through a public payer, and the presence of any extra-cardiac vascular or infectious complications of IE. ED: emergency department. MOUD: medication for opioid use disorder. Tdap: tetanus, diphtheria and pertussis vaccine

The results of our adjusted logistic regression analyses are shown in Fig. 1. Implementation of the IMAT program was not independently associated with changes in healthcare utilization, including both 30-day post-discharge hospital readmissions (aOR 0.50 CI[0.10–2.4]) and 90-day post-discharge ED visits (aOR 0.70 CI[0.23–2.1]). There were, however, differences in metrics for quality care. Vaccination rates for hepatitis A and Tdap were independently associated with the implementation of the IMAT program, with adjusted odds of 5.3 (CI: 1.2–32) and 9.2 (CI: 2.0–59), respectively. The adjusted odds of receiving prescriptions for MOUD and naloxone were 110 (CI: 16–1500) and 18(CI: 1.1 -1600), respectively, after implementation of the IMAT program.

## Discussion

Infective endocarditis is a major source of morbidity and mortality among infectious sequelae of the overdose crisis. Our study sheds light on the impact of one multidisciplinary addiction care intervention in the acute care setting seeking to minimize the morbidity of IDU-IE. We found that, compared to prior to the implementation of the IMAT program, when MOUD and other substance use care was managed by either the psychiatry consult liaison alone and/or primary admitting team, MOUD and naloxone prescribing and administration of hepatitis A and Tdap vaccines increased significantly. While not statistically significant in the regression analysis, hepatitis B vaccination rates also increased. These collective findings could be explained by multidisciplinary meetings and trainings between the IMAT team and infectious disease service, as well as the development of EHR tools and order sets to streamline medical management of IDU in the inpatient setting, including infectious disease screening and vaccinations. We also suspect that the creation of a multidisciplinary service to meet the medical needs of PWID increased rapport between patients and hospital providers, likely increasing patients’ participation in recommended interventions. We found that implementation of the IMAT program was not independently associated with changes in post-discharge healthcare utilization, including both 30-day hospital readmissions and 90-day ED visits, although 30-day readmission rates did decrease in descriptive analyses. Prior work has shown duration-dependent effects of MOUD treatment on healthcare utilization outcomes, with patients receiving MOUD for at least 6 months requiring less acute care utilization than patients receiving MOUD for less time [29]. This perspective offers one possible explanation for why acute care utilization in our study may not have changed within 30 or 90 days of IMAT intervention – such follow-up time frames may not be adequate to reflect maximal benefit from MOUD prescribing.

Together, these results suggest that inpatient addiction care during IDU-IE hospitalizations may not attenuate short-term healthcare utilization, but has the potential to improve the quality of care patients with IDU-IE receive while admitted (i.e. MOUD, naloxone, key vaccinations), and thus reduce long-term healthcare utilization and morbidity associated with IDU (i.e. recurrent IE related to repeated exposures, or infection with communicable illnesses). Indeed, one 2020 modeling study found that providing substance use rehabilitation services in conjunction with parenteral antibiotic treatment in the treatment of patients with IDU-IE requiring cardiac surgery would be cost effective [30].

While reducing expenditures associated with increased healthcare service utilization is one benefit of reducing the morbidity associated with IDU-IE, such benefits should not be unduly prioritized over the human experience of receiving and providing quality addiction care in the acute care setting. This study did not assess how receiving addiction care services mediated patients’ experiences of illness or attitudes toward the acute-care setting, but others have found that care teams’ understanding of substance use disorders and access to peer support services are perceived positively by hospitalized patients and staff alike [31–33]. Furthermore, inpatient multidisciplinary addiction consult teams have been shown to promote trust between hospitalized patients with co-occurring substance use disorders and physicians [34]. These qualitative and experiential factors may mediate some of the IMAT program’s impact in improving MOUD and vaccination rates among people with IDU-IE

This study should also be interpreted in context of its limitations. Our study was based at a single tertiary care center in a metropolitan region of a largely rural state, which is one of two major cardiac referral centers in the state. This limited the number of patients with IE eligible for inclusion in the study, and likely accounts for the wide confidence intervals observed for some variables in the adjusted odd ratios; we thus encourage readers to interpret estimates of adjusted odds ratios with caution. A further limitation is that, due to our study’s retrospective design, we cannot account for bias caused by unknown confounding factors. Additionally, we do not differentiate between patients receiving induction or maintenance MOUD during their IDU-IE hospitalization. We also note that our study population, with only one non-White patient with IDU-IE, may not be generalizable to many communities. However, conducting the study in this community also provides important information on the unique context of addiction care in the acute care setting in a rural state. Prior work has indeed shown that rural patients with IDU-IE are increasingly seeking care at metropolitan hospitals, and that PWID in rural areas face unique challenges in accessing harm-reduction services [7, 8]. Further work has found regional variations in the morbidity associated with IDU-IE, with co-infection with HIV or hepatitis C more likely to occur in the Northeast, even as increases in incidence are most prominent in other regions [3]. Together with our results here, this body of work suggests that while IDU-IE is a national problem affecting urban and rural geographies alike, there is need for interventions responsive to local needs presented by the overdose crisis.

We also note that this study did not query public health records for vaccination data. Assessment of baseline vaccination status was based on vaccination and serological records available in the EHR only. Thus it is possible that our study underestimates the number of patients up-to-date with key vaccinations at time of presentation to care for IDU-IE.

Additionally, while prior work has demonstrated people with IDU-IE to be younger and have fewer comorbidities than people with IE not associated with IDU, further analysis, particularly in larger studies, to elucidate the underlying comorbidities that may drive health-care utilization and mortality (i.e. using the Charleston Comorbidity Index) are needed [14, 35].

Ultimately, this study demonstrates the need for further research to understand the long-term impact of addiction medicine care in the inpatient setting, particularly among people with IDU-IE and in the rural context. Further examination of hospital-based interventions for IDU would facilitate the continued improvement and access to these programs, particularly in rural states like ours where patients face decreased access to post-discharge substance use disorder and harm reduction services [8].

## Conclusions

We found that the implementation of a multidisciplinary addiction care team at a tertiary care center in a rural state was independently associated with increased MOUD and naloxone prescribing and hepatitis A and Tdap vaccination among patients hospitalized for IDU-IE. Further studies are needed to characterize the short and long-term impacts of addiction care in the acute care setting on healthcare service utilization outcomes, and to understand the role in which rurality mediates the impact of these interventions.

## Electronic supplementary material

Below is the link to the electronic supplementary material.

Supplementary Material 1

Supplementary Material 2

## Data Availability

De-identified data may be made available upon request to the corresponding author with reasonable request. Data is not publicly available in order to protect patient privacy and reduce the risk of de-identification.

## Declarations

### Footnotes

No footnotes.

## Abbreviations

HIV: Human immunodeficiency virus
HBV: Hepatitis B virus
HCV: Hepatitis C virus
IE: Infective endocarditis
IDU-IE: Infective endocarditis associated with injection drug use
NIS: National (Nationwide) Inpatient Sample
Non-IDU-IE: infective endocarditis not associated with injection drug use
ED: Emergency department
PWID: people who inject drugs
MOUD: medication for opioid use disorder
MMC: Maine Medical Center
TEE: Transesophageal Echocardiogram
CVIS: Cardiovascular Information System
EHR: Electronic Health Record
PCP: Primary care provider
Tdap: Tetanus, Diphtheria and Pertussis
VIF: Variance Inflation Factor
