# Supplementary material for: Inpatient addiction care is associated with increased vaccinations, medication for opioid use disorder and naloxone prescribing among patients with infective endocarditis in a rural state
Source: Addict Sci Clin Pract. 2025 Oct 16;20:82. doi: 10.1186/s13722-025-00614-6 (PMC12533352; doi:10.1186/s13722-025-00614-6)
Supplement: Supplementary file 1 — Supplementary Material 1 [file 13722_2025_614_MOESM1_ESM.docx]

| SI Table A. Demographic and health characteristics of patients with infective endocarditis at a Tertiary Care Center in Maine, 01/2013-01/2019 | | | | |
| --- | --- | --- | --- | --- |
| **Characteristic** | **Overall***^1^* | **IDU***^1^* | **No IDU***^1^* | **p-value***^2^* |
| **n** | 193 | 99 | 94 |  |
| **Assigned female at birth** | 67 (35) | 39 (39) | 28 (30) | 0.2 |
| **Insurance** |  |  |  | <0.001 |
| Public insurance only | 115 (60) | 65 (66) | 50 (53) |  |
| Uninsured | 30 (16) | 28 (28) | 2 (2.1) |  |
| Private insurance only | 25 (13) | 4 (4.0) | 21 (22) |  |
| Both public and private insurance | 23 (12) | 2 (2.0) | 21 (22) |  |
| **Documented PCP***^3^* | 152 (79) | 67 (68) | 85 (90) | <0.001 |
| **Unhoused** | 29 (15) | 28 (28) | 1 (1.1) | <0.001 |
| **No mental health conditions** | 61 (32) | 1 (1.0) | 60 (64) | <0.001 |
| **History of Hepatitis C** |  |  |  | <0.001 |
| No Hep C screening done | 80 (41) | 10 (10) | 70 (74) |  |
| Positive Hep C test*^4^* | 70 (36) | 69 (70) | 1 (1.1) |  |
| Negative Hep C test | 43 (22) | 20 (20) | 23 (24) |  |
| **Comorbid alcohol use***^5^* | 30 (16) | 23 (23) | 7 (7.4) | 0.002 |
| *^1^* n (%); IDU = Injection Drug Use | | | | |
| *^2^* Pearson’s Chi-squared test or Fischer’s exact test | | | | |
| *^3^* Primary care provider (PCP) on file  *^4^* Viral load or serology  *^5^* Alcohol use disorder or “alcohol abuse” documented in patient’s chart | | | | |
