# Supplementary material for: Inpatient addiction care is associated with increased vaccinations, medication for opioid use disorder and naloxone prescribing among patients with infective endocarditis in a rural state
Source: Addict Sci Clin Pract. 2025 Oct 16;20:82. doi: 10.1186/s13722-025-00614-6 (PMC12533352; doi:10.1186/s13722-025-00614-6)
Supplement: Supplementary file 2 — Supplementary Material 2 [file 13722_2025_614_MOESM2_ESM.docx]

| SI Table B. Clinical characteristics of patients with infective endocarditis at Tertiary Care Center in Maine, 01/2013-01//2019 | | | | |
| --- | --- | --- | --- | --- |
| **Characteristic** | **Overall***^1^* | **IDU***^1^* | **No IDU***^1^* | **p-value***^2^* |
| **n** | 193 | 99 | 94 |  |
| **Definite Endocarditis***^3^* | 149 (77) | 88 (89) | 61 (65) | <0.001 |
| **Valve type** |  |  |  | 0.003 |
| Right-sided | 88 (46) | 50 (51) | 38 (40) |  |
| Left-sided/left and right-sided | 67 (35) | 39 (39) | 28 (30) |  |
| No definite valvular involvement | 38 (20) | 10 (10) | 28 (30) |  |
| **Native valve endocarditis** | 141 (73) | 86 (87) | 55 (59) | <0.001 |
| **Vascular phenomena***^4^* | 74 (38) | 50 (51) | 24 (26) | <0.001 |
| **Immunological phenomena***^5^* | 15 (7.8) | 9 (9.1) | 6 (6.4) | 0.5 |
| **Infectious complications***^6^* | 118 (61) | 73 (74) | 45 (48) | <0.001 |
| **Cardiac device***^7^* | 19 (9.8) | 4 (4.0) | 15 (16) | 0.005 |
| **Blood culture** |  |  |  | 0.8 |
| Gram-positive only | 157 (81) | 82 (83) | 75 (80) |  |
| Negative blood cultures | 20 (10) | 9 (9.1) | 11 (12) |  |
| Gram-negative only | 7 (3.6) | 3 (3.0) | 4 (4.3) |  |
| Yeast only | 5 (2.6) | 2 (2.0) | 3 (3.2) |  |
| Polymicrobial | 4 (2.1) | 3 (3.0) | 1 (1.1) |  |
| **Death during admission** | 24 (12) | 12 (12) | 12 (13) | 0.9 |
| **Documented 90-day mortality** | 5 (2.6) | 0 (0) | 5 (5.3) | 0.026 |
| *^1^* n (%); IDU= injection drug use | | | | |
| *^2^* Pearson’s Chi-squared test or Fisher’s exact test | | | | |
| *^3^* Versus possible endocarditis, by Duke’s criteria | | | | |
| *^4^* I.e. major arterial emboli, septic pulmonary infarcts, mycotic aneurysm, intracranial hemorrhage, conjunctival hemorrhages and/or Janeway lesions | | | | |
| *^5^* I.e. glomerulonephritis, Osler nodes, Roth spots and/or rheumatoid factor | | | | |
| *^6^* I.e. septic emboli, septic joint, osteomyelitis, skin/soft tissue infection and/or epidural abscess | | | | |

*^7^* I.e. pacer, automatic implantable cardioverter defibrillator
